# Distribution and fate of HIV-1 unintegrated DNA species: a comprehensive update

**DOI:** 10.1186/s12981-016-0127-6

**Published:** 2017-02-16

**Authors:** Faysal Bin Hamid, Jinsun Kim, Cha-Gyun Shin

**Affiliations:** Department of Systems Biotechnology, Chung-Ang University, Anseong, Kyunggido 456-756 South Korea

**Keywords:** Retrovirus, HIV-1, Nuclear import, 2-LTR, Unintegrated DNA

## Abstract

Reverse transcription of viral RNA and the subsequent integration of reverse transcripts are the classical early events of the HIV-1 life-cycle. Simultaneously, abundant unintegrated DNAs (uDNAs), are formed in cells ubiquitously. The uDNAs either undergo recombination or degradation or persist inactively for long periods in the nucleus as future resources. Among them, 2-LTR circles are considered a dead-end for viral spread. Their contribution to the HIV-1 infection is still poorly understood. Nevertheless, the preintegration transcription of the aberrant DNAs and the consequent alterations of cellular factors have already been reported. Since the major fate of the viral genome is to persist as episomal DNA, precise characterization is required for studying the biology of HIV-1. This review compiles the biochemical and genetic updates on uDNA in the HIV-1 life cycle and could provide direction to further study of their roles in HIV-1 replication and application in HIV-1 pathogenesis.

## Background

Conversion of single-stranded RNAs to linear double-stranded reverse transcripts and their subsequent integration into host chromosomes are signature features of the early stage of the human immunodeficiency virus (HIV) lifecycle [1, 2]. Reverse transcription occurs in the cytoplasm, and the translocation of cDNA to the nucleus gives rise to at least two types of cDNA: linear and circular. Linear cDNA is integrated into host DNA and is transcribed into viral mRNA used in making viral progeny. However, circular forms of viral DNA, containing either one or two copies of the long terminal repeat (LTR) region, accumulate in the infected cells because of abortive integration processes. Although different studies report that they can express a limited range of early genes [3–8], the role of these unintegrated DNAs is still unclear.

Retroviruses enter host cells by endocytosis or by binding to glycoproteins on their surface. Specifically, HIV-1 binds to the receptor CD4 and co-receptors CCR5 and CXCR4 on T lymphocytes and macrophages [9, 10]. After the reverse transcription of viral RNA, which occurs in the endocytosed viral core and uses free cytoplasmic nucleotides and ions, the viral envelope is gradually released in a process known as uncoating [11]. The viral reverse transcripts then bind to at least four viral proteins: matrix (MA), integrase (IN), reverse transcriptase (RT), viral protein (Vpr), and/or capsid (CA), as well as several host proteins. These form a ribonucleoprotein complex, called the pre-integration complex (PIC), which enters the nucleus [10, 12]. After nuclear import, the cDNA either becomes integerated, or fails to integrate and may become circularized to form episomal DNA [3, 13, 14]; circular episomal DNA has in some cases been found in the cytoplasm as well [15, 16]. Delelis et al. [15] hypothesized that in prototype foamy virus-infected cells, it may be either that the 2-LTR circles formed in the nucleus are unable to be exported into the cytoplasm or that they might be yielded in the cytoplasm and be imported into the nucleus and accumulated. Either of these two events can explain the higher amount of 2-LTR circles detected in the nucleus and their higher stability.

Although linear DNA is considered as a substrate for provirus production, its ends can also be ligated to form 2-LTR circles by host non-homologous DNA end joining (NHEJ), one of the cellular repair systems. Another form, 1-LTR circles, can result from defective reverse transcription [17], auto-integration from the rearrangement of circular forms [1, 18], or homologous recombination between 2-LTR circles [1]. Several proteins of NHEJ have been reported to be involved in this process, such as the Ku70/80 heterodimer, ligase 4, XRCC4, and RAD52 [18–20]. Another type of unintegrated DNA, also known as autointegrants, is formed through the ligation of the internal region of viral DNA to the IN-processed 3′-end [3, 21–24]. The presence of autointegrants has been confirmed in HIV-1, as well as in other retroviruses, e.g. Moloney murine leukemia virus (MoMLV) and Rous sarcoma virus (RSV) [1, 18, 21].

## Distribution

After entry into cells, the reverse transcription of HIV-1 RNA occurs in the cytoplasm. Once inside the cytoplasm, viral reverse transcripts form PICs with host proteins. These complexes containing viral double stranded DNA (dsDNA), which has the triple stranded DNA structure called DNA flap, are able to be integrated into the host dsDNA in vitro. This is mediated by IN, which can cleave the 3′-ends of both LTRs of the blunt (or unprocessed) DNA, named linear unintegrated DNA (uDNA_L_), and results in 3′-processed linear DNA (pDNA_L_) [25]. The cleavage is occurred by tetrameric IN in case of both palindromic LTR-LTR junction and 2-LTR circle internally [26]. The DNA in PICs may either undergo circularization or remain in the form of linear dsDNA. Munir et al. [27] updated the order of the persistence of viral DNA forms as follows: provirus > circular DNA (1-LTRc and 2-LTRc) > uDNA_L_ > pDNA_L_. The pDNA_L_ is less stable than uDNA_L_ and can therefore persist a shorter period than uDNA_L_. The HIV-1 infection causes the accumulation of unintegrated DNAs in any cell type or cellular status in vivo [8, 27–29] or in vitro [1, 30–32] (Table 1). Gene expression from uDNA is higher than integrated proviruses in non-dividing cells compared to dividing cells [14]. The reason is likely due to lack of dilution of uDNA templates, the transcripts and proteins [3, 14, 33]. Interestingly, several groups have shown that the unintegrated circular forms of HIV-1 are present prominently in the nucleus and can be considered as markers of the active transport of the PIC into the nucleus [3, 29, 34]. Avian sarcoma virus was first reported to form circular DNA in infected cells, particularly in the nucleus [35, 36] (Table 1). However, unintegrated DNA was first reported in brain and blood tissue of HIV-1 infected dementia patients [37]. Later, high levels of unintegrated DNA were found in HIV-1 infected cells, both in in vivo on human patients and animals [38, 39] and in in vitro experiments on lymphocytic and monocytic cell lines [1, 35]. This was expected, as the ratio of linear to 1-LTR circular to 2-LTR circular HIV DNA was previously approximated as 20:9:1 [40, 41]. Interestingly, 1-LTR circles appeared earlier; at approximately at 2 h post infection (hpi) in HIV-1 infected MT-2 cell lines, while 2-LTR circles were found at about 12 hpi [35]. However, the 2-LTR circles were detected at 1–2 h hpi in the cytoplasm of murine leukemia virus (MLV)-infected cells [16] and after 72 hpi in prototype foamy virus-infected cells, although they started to accumulate already at 3 hpi in the infected cells [15]. Initially, it was thought that this was due to the lack of cell division in aphidicolin-treated NIH 3T3 cells, but 2-LTR circles also formed in arrested and non-arrested TE671 human medulloblastoma cells and ARPE-19 human retinal epithelial cells [17] (Table 1). It is considered that 1-LTR circles are more abundant than 2-LTR in infected cells both in vitro and in vivo [42, 43]. The relative abundance is cytoplasmic linear DNA > proviruses > 1-LTR circles > 2-LTR circles [42]. However, the autointegration of the HIV-1 genome produces cDNA products either containing nicked, inverted, or modified dsDNA circles [43]. This cytoplasmic DNA is detected by DNA sensors and secreted interferons [23, 44].

**Table 1:** Examples of unintegrated DNAs in virus-infected cells and their fates

Virus	Cell line	Cell number	hpi (hours per infection)	Methods	Consequence		Ref.
Avian sarcoma virus	QT-6		5 hpi		Closed circular DNA		[34]
MLV	NIH 3T3, TE671, ARPE-19	1 × 10^6^ cells	2 hpi	qPCR	2-LTR		[16]
HIV-1	MT-2	5 × 10^5^ cells	2 hpi	PCR and Southern hybridization	1-LTR		[31]
			12 hpi		2-LTR		
	Monocyte	1 × 10^6^ cells	2 hpi	PCR and Southern hybridization	1-LTR		
			12 hpi		2-LTR		
	Macrophage	3 × 10^5^ cells	4 dpi	qPCR	2-LTR	30–35 copies/100cells	[45]
			21 dpi			6 copies/100cells	
	CD4+ T lymphocytes	3 × 10^5^ cells	4 dpi		2-LTR	11 copies/100cells	
			14 dpi			X	
	Jurkat	1 × 10^7^	3 dpt		2-LTR	8.97%	[36]
					1-LTR	46.21%	
	293T				2-LTR	5.42%	
					1-LTR	40.16%	
	SupT1 (lymphoid cells)	5 × 10^6^ cells	15 hpi		2-LTR	0.01 copy/cell	[46]
			12 hpi			40 copies/cells	

*dpt* days post-transduction, *dpi* days post infection

In the infected cells, non-integrating HIV-1 and HIV-1-based vectors are organized into chromatin structures and enriched with histone modifications typical of silenced chromatins that can be reactivated upon exposure to histone deacetylase (HDAC) inhibitors. [47]. Surprisingly, 1-LTR circles are formed earlier than 2-LTR ones [27]. However, a significant amount, i.e. around 10%, of 1-LTR circles is formed during reverse transcription in the cytoplasm [14, 27]. Kilzer and his colleagues demonstrated that a high level of 1-LTR circles (90%) is generated after nuclear import by homologous recombination between two flanked LTR regions of linear DNA and that most of the 1-LTR circles are found in the nucleus [13]. The mechanism of 1-LTR circle formation and the sub-cellular localization points at its plausible significance in viral replication, mainly in the step of viral integration [27]. The strand-transfer inhibitor raltegravir (RAL) and the catalytic mutation of IN affected 3′-LTR processing differently: the comparison of 2-LTR circle accumulation mediated by either indicated that 3′-end processing has no role in 2-LTR circle accumulation, while the type of the LTR–LTR junction formation is considerably affected. The formation of 2-LTR circles containing original palindromic sequence junctions (40%) in RAL-treated cells were similar to that in wild type cells, but was nearly doubled in D116N mutants [27]. The pattern of 3′-end processing in circular DNA could be useful in designing the IN-targeting therapeutic agents.

### Role of cellular proteins in unintegrated DNA formation

Integration is required for successful retroviral replication. Cellular proteins RAD50, MRE11, and NBS1 are nuclease components that reportedly participate in 1-LTR circle formation [3, 13]. DNA-dependent protein kinase (DNA-PK), a nuclear serine-threonine protein kinase that can phosphorylate downstream proteins after sensing DNA breaks, is essential in the repair of dsDNA breaks by NHEJ. It is composed of a DNA-binding Ku70/Ku86 heterodimer and a 469 kDa catalytic subunit, DNA-PK_cs_ [48]. It has a significant role in V(D)J recombination as well. The RAG1/RAG2 proteins generate breaks in the strands of dsDNA. At the break-sites, the Ku70/Ku80 heterodimer binds to the free DNA termini, keeping them localized. DNA-PK_cs_ binds the DNA-bound Ku to form the DNA-PK complex, which stimulates DNA-PK_cs_ activity through phosphorylation [49]. It is known that 2-LTR circles are products of DNA repair mechanisms in the nucleus as a host reaction to foreign dsDNA [50]. Viral cDNA replication intermediates have been found to interact with host Ku components of the NHEJ pathway [51, 52]. Silencing of the NHEJ components Ku, ligase 4, or XRCC4 decreases the number of 2-LTR circles. Interestingly, the absence of DNA-PKcs, a component of the NHEJ machinery, showed the opposite effect [13, 52].

## Fate

### Degradation

**Fig. 1:**
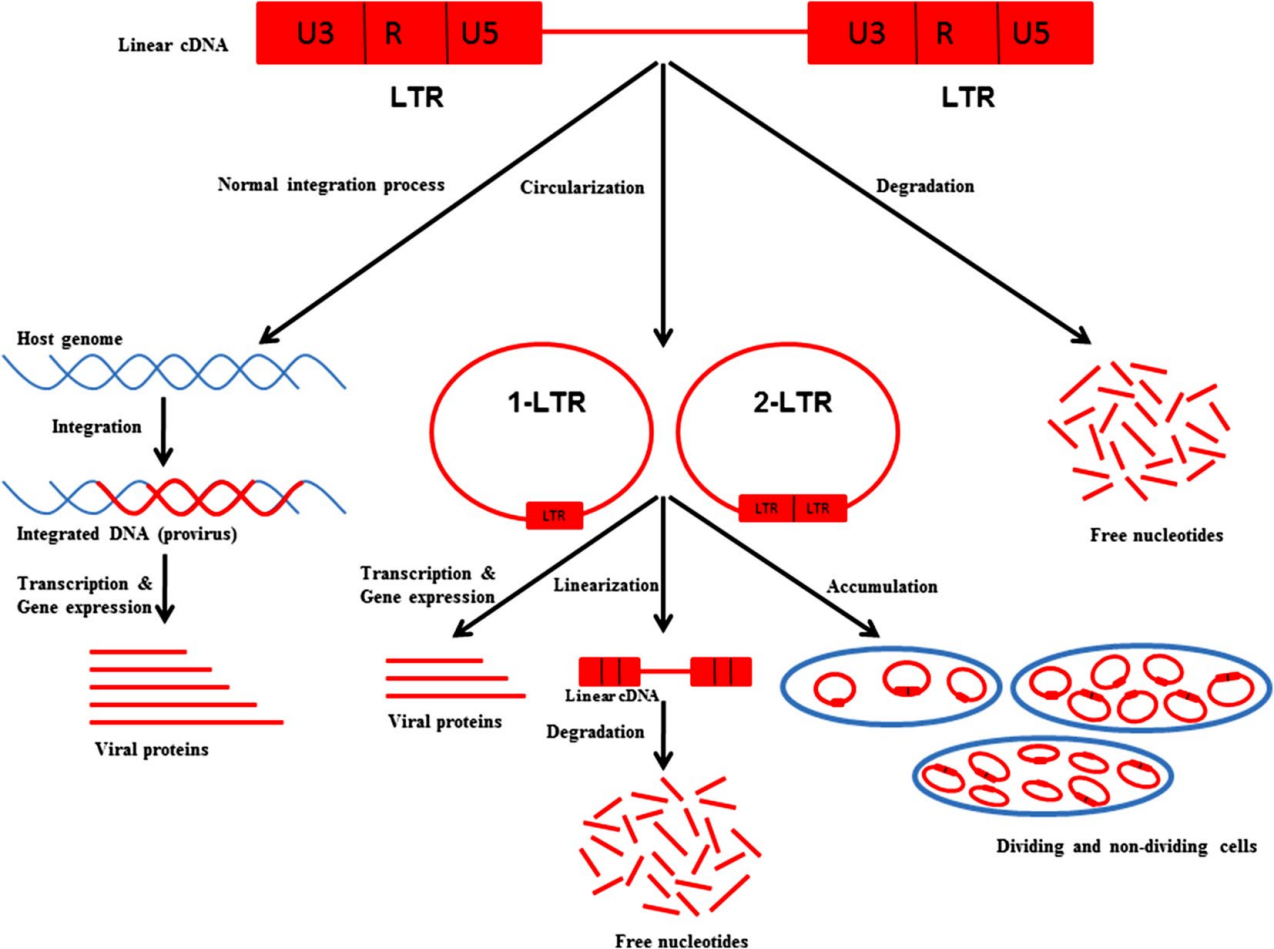
Fates of viral cDNAs (reverse transcripts) after reverse transcription. After reverse transcription, HIV-1 produces cDNAs that may either be integrated into host genome or circulate themselves. Otherwise, they are degraded into free nucleotides. Circular DNAs have been reported to actively participate in current gene expression similar to the integrated DNAs followed by linearization, degradation, or accumulation as a reservoir

Circular DNAs are considered as replicative dead end. Linear DNA, the substrate of integration, is degraded in dividing T cells within hours [53], but persists for several days in resting T cells; it may even persist up to one month in macrophages [8, 54, 55] (Fig. 1). In the pre-integration state, viruses express their genes and produce half of the rescuable virions in resting CD4+ T cells [55]. The shortage of the virus production may be due to the virus-induced programmed cell death (apoptosis) or blockage of reverse transcription. In addition to these fates, in the high-titer infections of cultured cells, the majority of viral DNA undergoes intracellular degradation after synthesis [41, 46, 56, 57]. Several further proposals have also been made for DNA repair factors acting on retroviral DNA [58, 59].

Highly active antiretroviral therapy (HAART) causes a dramatic reduction in HIV-1 replication during the first 3 years and lowers the level of 2-LTR circles as well [60]. Furthermore, after 7–8 years of uninterrupted therapy, 2-LTR circles were almost undetectable in peripheral blood mononuclear cells (PBMCs) of most of the patients. These observations indicate the labile nature of 2-LTR circles, which may be subject to continuous HAART therapy. However, HIV-1 DNA was still detectable in the infected patients [60], probably due to the ongoing viral replication in newly infected dividing cells. Previously, it was demonstrated that the gradual decrease of the amount of circular DNA in proliferating cells occurs due to the absence of an origin of replication and is a function of dilution resulting from cell division [53]. IN mutant-infected macrophages produce DNA that persists at low levels even after 2 weeks, most of which is presumably unintegrated DNA. It was confirmed that the stability of 2-LTR was not due to viral replication [61].

### Reservoirs

Latent unintegrated genomes present in viremic HIV-1 patients after HAART treatment act as reservoirs and are an obstacle to effective treatment. 2-LTR circles persist in HIV-1 infected, growth-arrested T cell lines beyond their estimated half-life as previously determined in dividing cell populations [45, 56]. The drop of 2-LTR circle levels in dividing cells might be due to cell division, not due to degradation [54, 62]. In non-dividing macrophages, the 2-LTR circle levels remained the same for up to 21 days post infection (dpi) (Fig. 1). The unvarying 2-LTR circle level indicates their high stability in macrophages [45]. Moreover, macrophages are resistant to cytopathic effects caused by HIV-1, which makes macrophages a suitable reservoir for further infection [8, 33, 45, 62]. This stability in non-replicating cells may be due to their association with host proteins, e.g. histones, which protects them from decay [62]. E-DNA would be diluted out with successive rounds of cell division. In contrast to highly-active antiretroviral therapy (HAART)-treated HIV-1 patients, elite controllers, a group of HIV-1 infected persons who can control HIV-1 replication naturally in the absence of antiretroviral therapy and maintain untraceable viral loads, had higher 2-LTR circles than integrated proviruses in Ex Vivo experiments [63, 64].

### Gene expression

The integrated HIV-1 provirus is transcribed into new genomic RNA, also serving as mRNA, which is in turn translated into viral proteins [2–6, 65] (Fig. 1). Although it is speculated that the integrated copies of viral DNA are the sole template for viral gene expression, there is also evidence of preintegration transcription from unintegrated DNA [5, 65] (Fig. 1). Most recently, it has been shown that the transcriptional interplay is regulated oppositely between integrated and unintegrated DNA following NF-κB pathway modulation [66]. Upon various pharmacological treatments of NF-κB pathway activation, transcription factors such as NF-κB p65 and AP-1 (cFos/cJun) binds to integrated DNA and increases its expression, though the uDNA expression is declined. On the other hand, inhibition of the NF-κB pathway supports the expression of circular uDNA, and Bcl-3 and AP-1 is associated with its LTR region [66]. However, the persistent expression of HIV-1 proteins has already been reported not only in vitro in non-dividing cells, such as primary rat neurons and growth arrested fibroblasts, and dividing cells, such as SV40 T-antigen expressing cells [67–70], but also in vivo in rodent ocular and brain tissues [69]. Even IN-mutant HIV-1 or integration-arrested viruses produce transcriptionally active circular DNA [4, 5, 67, 71]. It is already established that HIV-1 circular unintegrated DNA can promote viral replication itself, but several groups disapproved the notion [40, 47]. Most recently, Shimura et al. [72] reported that the expression of circular DNA genes is possible even after IN strand-transfer inhibitor treatment, probably due to cell-to-cell infection. Particularly, 2-LTR circles can express not only early viral genes, such as *Nef* [5, 65, 72], *Tat* [40, 73], and *Rev* [5, 40, 72], but also late, non-spliced, singly- or multiply-spliced transcripts prior to integration. Among them, the *Nef* and *Tat* proteins are translated only from the fully spliced mRNA [4]. It has been reported that the expression of circular DNA genes, especially *Nef*, is augmented by *Vpr* in HIV-1 infected cells [74]. Interestingly, *Nef* can stimulate T cell activation and decrease the expression of co-receptors CD4, CXCR4, and CCR5, thus increasing HIV-1 infectivity [75]. In addition, *Tat*, a transactivator protein of HIV-1, can activate the transcription from LTRs of both unintegrated and integrated viral DNA [76]. Particularly, viral transcripts are expressed from the upstream promoter, probably from the beginning of the R region located within the tandem LTR repeats of a 2-LTR circle [72]. In non-dividing cells, such as macrophages, gene expression is induced by *Vpr* only when it is driven by the HIV-1 LTR promoter, but not by the cytomegalovirus promoter [45]. However, after integration, Rev activity increases in order to support the production of late genes, such as *Vpu*, both from spliced and unspliced genes [5, 75]. Recently, Emeagwali and his colleagues (2012) showed that *Vpu* and the antagonistic host protein TWIK-related acid sensitive K+ channel 1 (TASK1) can preferentially downregulate the transcription of episomal DNA [75]. However, a greater part of nonintegrated viral DNA might be inactive templates for the transcription machinery. When it was observed experimentally, the *Rev* transcripts were synthesized from nonintegrated DNA, although the expression level was not high (approximately 0.03–5% of total viral DNA) and was transient. Inadequate *Rev* expression was likely the result of the activity of the late structural gene *gag* in nonintegrated HIV-1 DNA, which was similar to IN defective virus-infected, inhibitor-treated, or quiescent cells [5]. Upon latency reversing agents including PKC activators, histone deacetylase inhibitors and P-TEFb agonists) treatment, latent uDNA was reported to initiate lately virus production as well as the latent integrated proviruses [14].

Surprisingly, 2-LTR circles can be used as a substrate for integration by IN and contribute to the spumaviral lifecycle, even after integration [77]. Previously, spleen necrosis virus (SNV), Rous sarcoma virus (RSV), avian sarcoma virus (ASV), and avian leukosis virus (ALV) were assumed to generate the LTR–LTR circles that are used as templates for integration [19, 77]. Generally, IN can cleave the viral double stranded cDNA ends in a staggered manner, which then undergoes covalent transesterification to the 5′ phosphates of the host dsDNA [2, 21]. However, IN also shows the novel pleiotropic action in the mechanism underlying the requirement in the circular DNA integration: it can directly cleave the conserved palindromic sequence found at LTR–LTR junctions and produce linear DNA from 2-LTR circles [77]. Most recently, it has been reported that HIV-1 PIC, specifically IN, can also cleave 2-LTR circles in a similar way [78]. HIV-1 IN required for linearization of 2-LTR circles was found at the palindromic junction, recognized as integration site, and subsequently executed a de novo integration process. It can explain the decrease in the amount of 2-LTR circles and surge of linear DNA, as well as proviral DNA, after the withdrawal of raltegravir in vitro [78] or HAART administration in vivo [39].

Despite the expression level of circular DNA that was reported previously, improved efficiency was observed in the long U3 deletion mutants, both in vivo and in vitro [32]. The U3-region truncation does not alter the diversity of the four types of episomal DNA. Interestingly, the U3 deletion causes high transgene expression from episomes in different levels in non-dividing brain cells and slowly dividing liver cells of rats. The effect of the large U3 deletion on episomal expression indicates that the *cis*-acting elements of the retroviral genome can regulate the extrachromosomal transcriptional activity and that the cell-specific *trans*-acting factors are presumably implicated in negatively regulated transgene expression from lentiviral episomes [32]. Moreover, the addition of HDAC inhibitors in the form of short-chain fatty acids can also induce gene expression, as well as replication, from episomal DNA. To crosstalk genetically and functionally between integrated and unintegrated DNA, it has been found that HIV-1 gene expression, such as of *Vpr*, from unintegrated DNA can be complemented by co-infection with the integrated viral genome [47, 79].

### Cytocidal effect on host cells

The cytocidal effect of retroviruses is thought to exist due to the rapid accumulation of unintegrated viral DNA in the cells. The reinfection property might be the reason of cytopathogenesis. Most acutely and chronically infected cells show the cytocidal effect [35]. A lower level of unintegrated DNA indicates that the cells are resistant to superinfection and maintain a persistent infection [35].

### Measurement of the HIV-1 reservior and its eradication

The durable nature of HIV-1 reservoir in all CD4+ T-cell subsets, including naïve and memory cells is a key problem to eradication. The establishment of the pool starts from the very initial time of primary infection associated with cytopathic effect and rounds of new infection. CD4+ T cells are reported to maintain high levels of HIV DNA even after HAART and it can resume high levels of new replication following ART withdrawal [64, 80]. However, the samples, the analytes, and the assays must be taken into consideration during the measurement of the reservoir. To date, several assays have been used to measure the different analytes which are considered as the HIV reservoirs (Table 2), but shortage of standardization and validation have made the assessment difficult. The problem of all PCR-based assays is that they can not discriminate between the infectious virion producing cells and latent viral reservoir containg cells (Table 2) [80]. So different real time PCRs have been used to measure the analytes such as viral RNA, 2-LTR and proviruses more accurately. But they are still lack of fidelity of quality and accuracy. Some of the current techniques used to measure the analytes that reflect the HIV-1 reservior have been discussed in Table 2.

**Table 2:** Different assays used to measure different analytes

Analytes	Assays	Advantages	Problems	References
Resting CD4+ T cells	PCR	Considered as ‘gold-standard’ for measuring latently infected cells	Low accuracy, slow, costly	[28, 80]
Total DNA	qPCR	Integrated and unintegrated HIV-DNA per million in peripheral blood mononuclear cells (PBMC) or CD4+ T cells	Unknown	[80]
Total HIV-DNA and 2-LTR circles	Droplet digital PCR	More sensitive and precise comparing to real time *pol* PCR	Short life span of 2-LTR; low accuracy	[80]
Provirus	*Alu*–*gag* PCR and qPCR	Conventional method to detect integrated proviruses.	Nonspecific, low accuracy	[80]
Intracellular HIV-RNA	PCR	Detection of 1 copy per million resting CD4+ T cells	Unknown	[80]
Viral RNA	HIV-RNA single copy assay (SCA)	Ultrasensitive method to quantify HIV-RNA in plasma	Unknown	[80]

## Conclusions

The 2-LTR circles are formed at the initial period of infection in vivo. Most of the cells undergo cell death, while some survive [53] and remain viral reservoirs. The longevity of these cells is due to the “intrinsic stability”, which is not identical in all cell types. The viral infection may induce cell survival factors, but the complete molecular mechanisms are still unknown. 2-LTR circles are a clear indication of foregoing viral replication, and the question of using them as valid markers of nuclear import for real time measurement arises [53, 81]. Many groups have reported on the significance of 2-LTR circle gene expression [3, 4, 7, 8], while the mechanisms of formation and gene expression and the fate of 1-LTR is in most cases omitted. The nuclear import of HIV-1 is not dependent on any particular viral or cellular karyophilic proteins [10, 82]. Since the study of the nuclear import and 2-LTR circles are closely related, the roles of these proteins should be observed more carefully. The complete characterization of the uDNAs might be convenient to understanding the HIV-1 viremia better. Moreover, the pleotropic fate of uDNA renders it a more eligible candidate for gene therapy and drug delivery.

## Abbreviations

uDNAs: unintegrated DNAs
HIV: human immunodeficiency virus
LTR: long terminal repeat
MA: matrix
IN: integrase
RT: reverse transcriptase
Vpr: viral protein
CA: capsid
PIC: pre-integration complex
MoMLV: Moloney murine leukemia virus
RSV: Rous sarcoma virus
uDNAL: linear unintegrated DNA
pDNAL: 3′-processed linear DNA
hpi: post infection
MLV: Murine leukemia virus
RAL: raltegravir
DNA-PK: DNA-dependent protein kinase
HAART: highly active antiretroviral therapy
PBMCs: peripheral blood mononuclear cells
dpi: days post infection
TASK1: TWIK-related acid sensitive K+ channel 1
